# Pemphigus vulgaris with refractory gingival ulcerations, successfully treated with rituximab—A case report

**DOI:** 10.1002/ccr3.6321

**Published:** 2022-09-14

**Authors:** Mateja Dolenc‐Voljč, Katja Povšič, Alja Cmok Kučič, Rok Gašperšič

**Affiliations:** ^1^ Department of Dermatovenereology, Faculty of Medicine University of Ljubljana Ljubljana Slovenia; ^2^ Department of Oral Medicine and Periodontology, Faculty of Medicine University of Ljubljana Ljubljana Slovenia

**Keywords:** desquamative gingivitis, gingiva, pemphigus vulgaris, rituximab, ulceration

## Abstract

A patient presented with ulcerations of the buccal mucosae, palate and gingiva. A gingival biopsy confirmed the diagnosis as pemphigus vulgaris. Despite medication with systemic corticosteroids and mycophenolate mofetil, desquamative gingivitis persisted. Adjunct treatment with rituximab was therefore introduced. Regular follow‐ups revealed no inflammatory gingival changes even 6 years later.

## Graphical abstract

Gingival lesions are considered one of the most resistant sites of pemphigus vulgaris. Their management, aimed at long‐term complete clinical remission, may include the use of rituximab in combination with systemic immunosuppressive drugs.

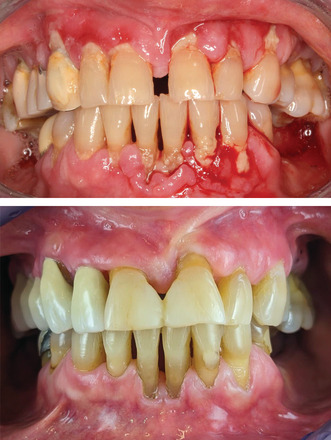

## 1 INTRODUCTION

Pemphigus vulgaris (PV) is a chronic autoimmune bullous disease induced by IgG autoantibodies against desmoglein 3, typically presenting with blisters, erosions, and ulcers of the skin and/or mucous membranes due to intraepidermal acantholysis.^1^ Lesions of the oral cavity, characterized by pseudo‐membranes covering painful ulcers, frequently appear on the soft palate, buccae, lips, tongue, and/or gingiva. Conventional treatment of oral PV includes the combination of systemic corticosteroids and additional immunosuppressive drugs, whereby some patients undergo severe side effects, relapses or are found to be non‐responders. Rituximab (RTX), a monoclonal antibody directed against the CD20 membrane proteins of B‐lymphocytes, has been proposed as a novel and safe treatment option.^2^ However, its effectiveness against gingival PV lesions has not yet been evaluated.

## 2 CLINICAL PRESENTATION

A 57‐year‐old female patient presented with persistent extensive ulcers of the oral cavity affecting the buccal mucosae, palate, and gingiva, which had first appeared 8 months ago. She complained of difficulties eating solids, speaking, and toothbrushing. She had also noticed an isolated blister on her left forearm as well as eye redness. She was a non‐smoker and non‐drinker; her family and personal medical history were non‐contributory.

**FIGURE 1:**
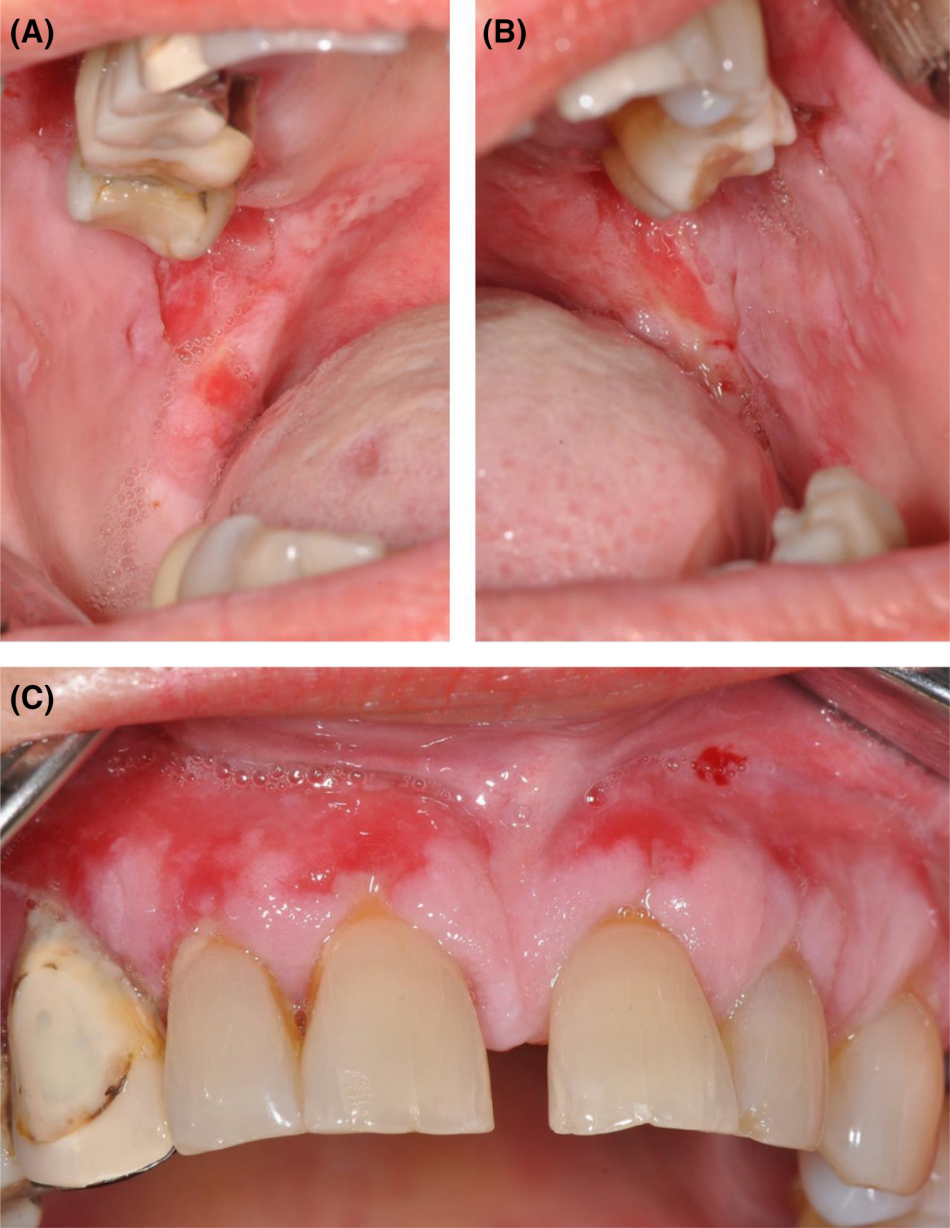
(A, B) Intra‐oral photograph showing bilateral ulcerative lesions on the buccal mucosa along the occlusal plane; lesions with irregular borders were associated with flaccid bullae in the molar region (A—right side; B—left side). (C) Intraoral photograph showing the erythematous attached gingiva with several erosions extending up to the free gingival margin

**FIGURE 2:**
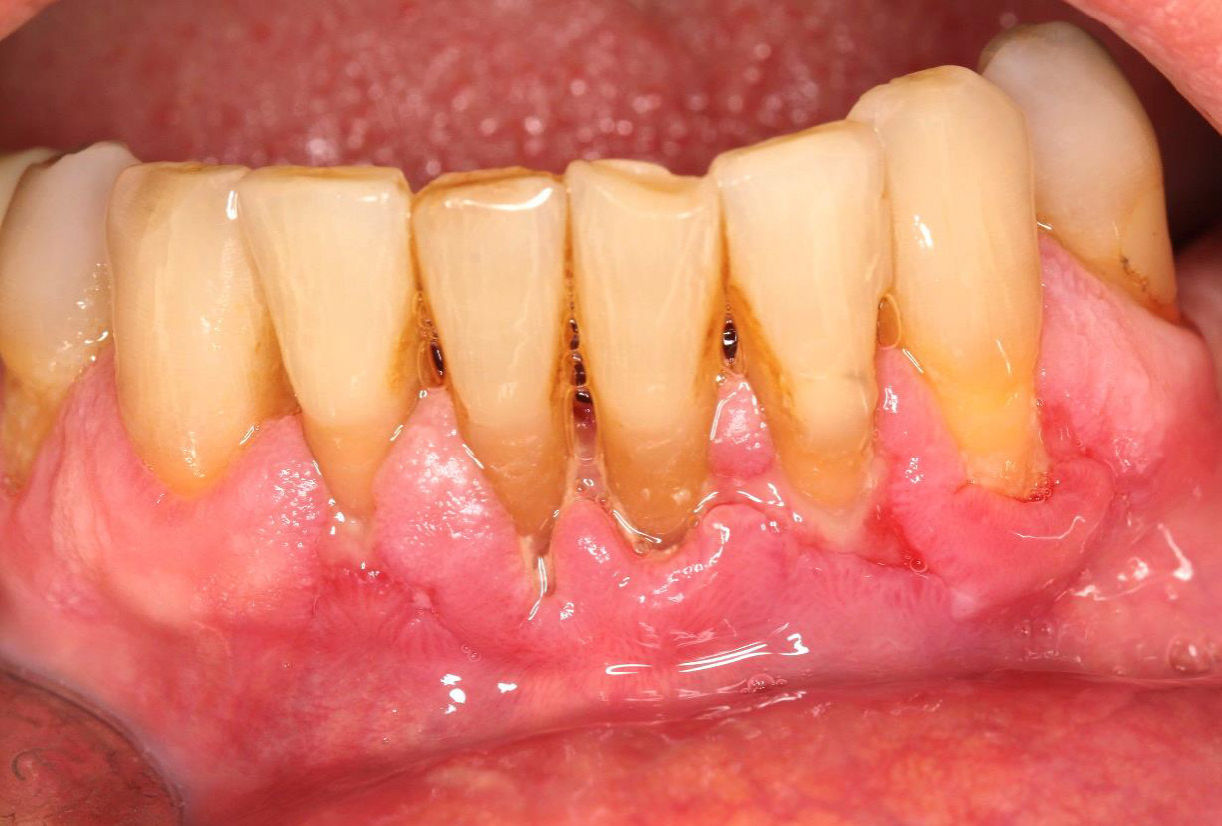
Attachment loss in the mandibular dental arch due to chronic periodontitis

An intraoral examination revealed bilateral ulcerative lesions of the buccal mucosae, extending from the glossopalatine arch and retromolar areas to the molar region along the occlusal plane (Figure 1A,B). They were irregular in shape and covered by pseudo‐membranes with erythematous halos. The erosive lesions also involved the attached gingiva (Figure 1C). In addition, the patient suffered from chronic periodontitis (Figure 2).

## 3 CASE MANAGEMENT

### 3.1 Diagnosis

An incisional biopsy was performed at the perilesional region of the attached gingiva above the left upper canine. Histopathological examination found supra‐basal acantholysis and chronic mononuclear lymphoplasmacytic inflammatory cell infiltrate in the subepithelial and perivascular regions. Direct immunofluorescence revealed intercellular deposits of IgG and C3 confined to the lower epithelial layers. Indirect immunofluorescence of the patient's serum showed desmoglein 3‐reactive autoantibodies, confirming the diagnosis of PV.

### 3.2 Treatment

In July 2012, treatment with methylprednisolone 52 mg/day and azathioprine 100 mg/day was initiated. Triamcinolone acetonide gel 0.1% was prescribed for the oral lesions. Motivation and instruction in correct oral hygiene using soft brushes was performed in addition to nonsurgical periodontal therapy: supra‐ and sub‐gingival plaque was removed using piezoelectric ultrasonic instruments (PiezoLED ultrasonic scaler with Piezo Scaler tip 203 [KaVo dental, Biberach, Germany]) under local anesthesia (Ultracain©, Hoechst, France). After 1 month, azathioprine was discontinued due to elevated liver enzymes; MMF 2 g/day was prescribed instead.

**FIGURE 3:**
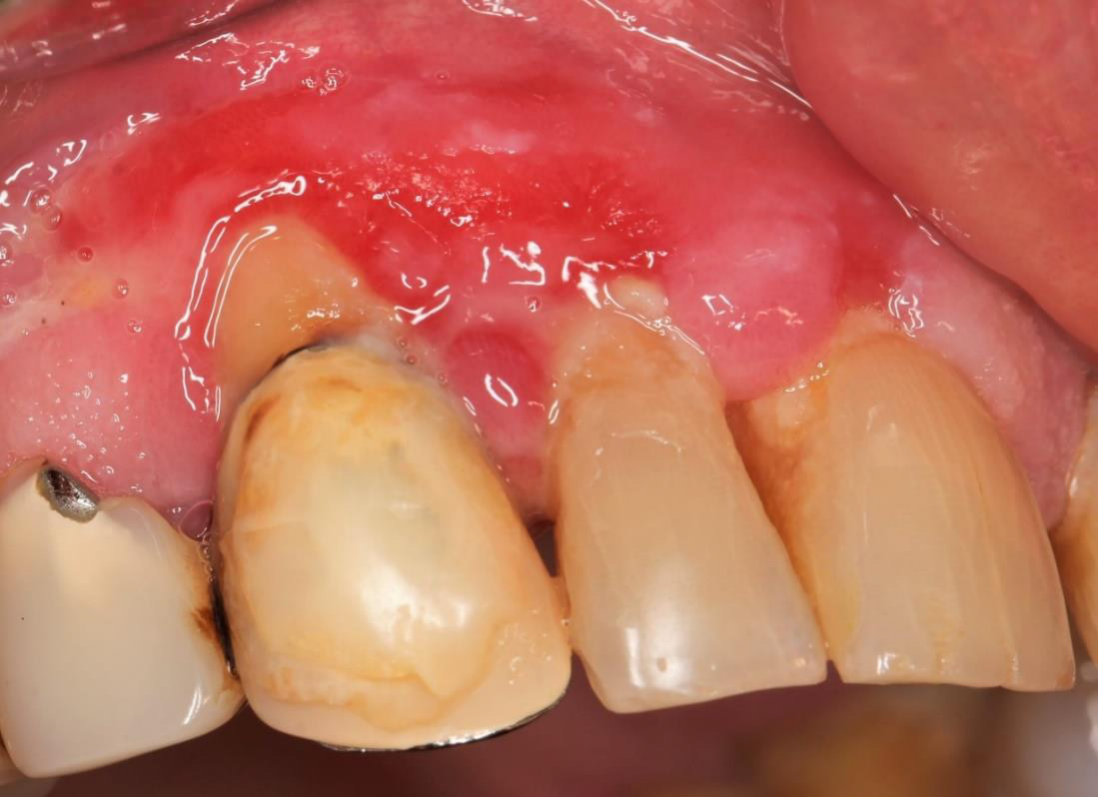
Recurrence of gingival ulcerations above the right maxillary incisors and canines

The patient made a slow but gradual recovery over the next 5 months, attending regular follow‐up visits every 3 months. No recurrences were observed until 2014, when painful gingival ulcerations covered by fibrinous exudates reappeared above the maxillary incisors and canines (Figure 3). The removal of supra‐ and sub‐gingival plaque was performed once again. In addition, dexamethasone 1 mg/ml oral rinse 3×/day was prescribed, leading to partial improvement. However, the gingival ulcerations persisted. Small erosions of the dorsal palate and buccal mucosae also reappeared over the following months. The patient suffered from weight loss due to pain in the oral cavity while eating.

**FIGURE 4:**
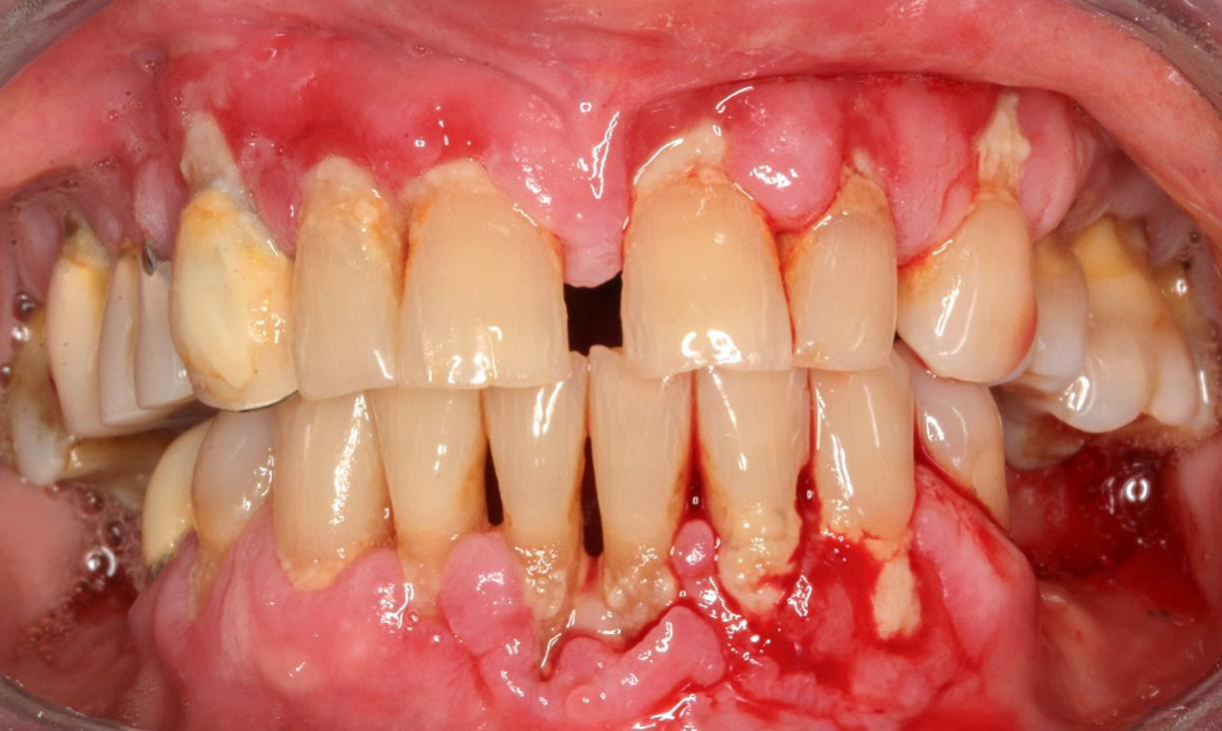
Resistance of the gingival ulcerations to conventional immunosuppressive treatment

Since the gingival ulcerations did not respond to conventional immunosuppressive treatment (Figure 4), the patient received 4 infusions of RTX (375 mg/m^2^ weekly) in July 2015. The daily doses of methylprednisolone and MMF were slowly tapered over the next weeks.

## 4 CLINICAL OUTCOMES

A remission of the gingival and mucous ulcerations was observed within the following months—the ulcerations gradually became smaller, less frequent, and non‐painful, showing adequate epithelization. Prosthetic rehabilitation was performed.

Over the next 2 years, however, depletion of B‐lymphocytes was observed, and the patient experienced recurring outbreaks of genital herpes.

**FIGURE 5:**
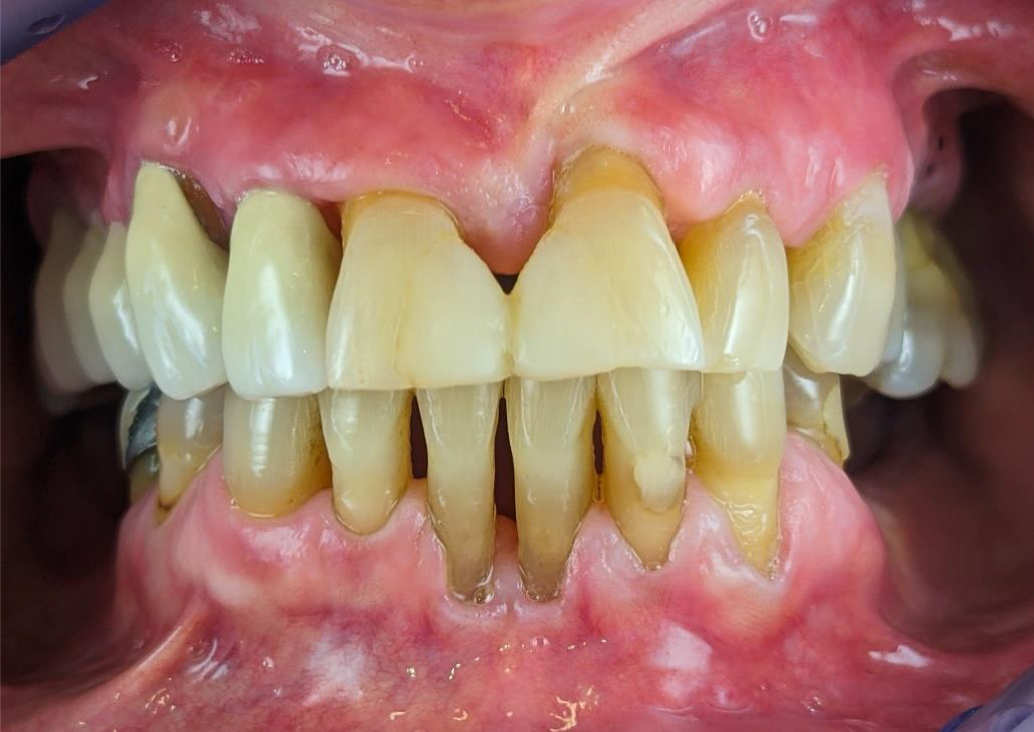
Intra‐oral photograph 6 years after treatment with RTXfrontal view

**FIGURE 6:**
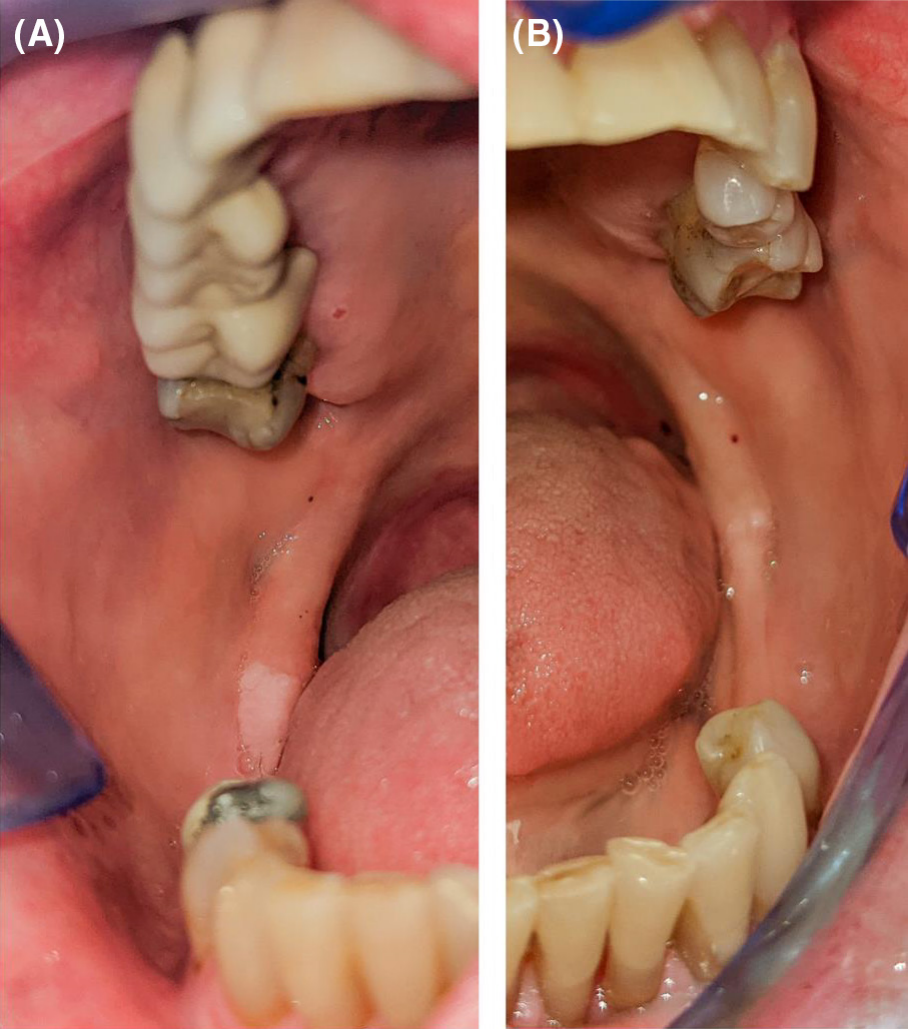
Intra‐oral photographs 6 years after treatment with RTX: (A) right retromolar area; (B) left retromolar area

In February 2017, she complained of dysgeusia and xerostomia. Five months later, a large erosion appeared on the mucosa of her upper lip and several others on the buccal mucosae. She was once again treated with a higher dose of systemic methylprednisolone (16 mg/day). In addition, 0.1% triamcinolone acetonide was prescribed for topical use. Since the mucosal lesions persisted, the patient received 5 cycles of intravenous immunoglobulins, 1 cycle with 2 g/kg monthly. Besides two small ulcers treated by local measures, regular follow‐ups have revealed no inflammatory changes of the gingiva even 6 years after RTX administration in conjunction with low maintenance doses of methylprednisolone and MMF (Figures 5 and 6).

## 5 DISCUSSION

The combination of systemic corticosteroids and other immunosuppressives has long been the established treatment of PV^3^ and was selected as the first‐line medication for our patient. However, it did not prevent the recurrence of oral lesions, indicating the need for second‐line treatment. RTX was chosen as an adjunct to methylprednisolone and MMF based on previous reports regarding its benefits in treating PV,^4^, ^5^, ^6^ including oral PV:^2^, ^7^ safety, mild transient side effects, low relapse, and high remission rate. RTX helped attain a lower dosage of the adjunct immunosuppressive drugs^8^ even though the onset of its action was delayed.^9^ Due to its many advantages, RTX has recently been recommended as a first‐line treatment in PV cases.^10^, ^11^ At the time our patient was treated with RTX, no standard protocol for its use existed. It was therefore chosen as an off‐label medication following the standard lymphoma protocol (4 weekly infusions).^6^, ^12^, ^13^ According to the new treatment algorithm of PV with RTX, two infusions of 1 g (administered 2 weeks apart, either alone or in combination with systemic corticosteroids) are recommended nowadays.^10^ Early treatment with RTX (≤6 months after diagnosis) may show better outcomes than late treatment (>6 months after diagnosis), since it is associated with a higher rate of complete remission, longer lasting remission phase, a lower number of adjuvants and a greater improvement of clinical effects.^14^ The need for regular maintenance therapy with RTX, usually proposed in intervals between 6–12 months, however, remains an unsettled subject since the risk/benefit ratio is yet unknown.^14^ It should also be noted that gingival PV lesions can be non‐anti‐Dsg1/3 dependent in some cases, whereby patients are found to be non‐responders to treatment with rituximab.^7^

After complete clinical remission, relapses subsequently appeared in the form of erosions on the patient's oral mucosa. However, no pathological changes were observed on the gingiva, even though it is considered one of the most resistant sites of PV.^15^ This may have occurred due to the combination of RTX and immunosuppressive drugs as well as the patient's improved hygiene practices. Namely, professional instruction in oral hygiene aimed at plaque control has been found to reduce clinical gingival inflammation and improve patient related outcomes, which alleviate gingival lesions and pain in PV patients.^16^, ^17^

The depletion of B‐lymphocytes is a well‐known side effect of RTX; it is a consequence of antibody‐dependent cell‐mediated cytotoxicity of RTX, complement‐mediated cytotoxicity and inhibited cell proliferation with direct induction of B‐cell apoptosis.^8^ This leads to a lowered production of autoantibodies yet may also predispose patients to other infections as a result of prolonged immunosuppression.^9^ In our patient, recurrent genital herpes was observed.

To conclude, desquamative gingivitis was the predominant clinical manifestation of oral PV in our patient, with mild involvement of the skin and conjunctiva. In contrast to a previous report,^15^ RTX medication successfully treated our patient's gingival PV lesions. According to the new treatment guidelines,^10^ RTX can be recommended as a first‐line treatment of oral PV even in the early stages of the disease.

## AUTHOR CONTRIBUTIONS

MDV and RG participated in the initial patient examination and diagnosis as well as conception of the paper. MDV planned and implemented the systemic treatment protocols. RG executed the surgical procedures and was in charge of oral care. ACK and KP performed the follow‐ups, analyzed the clinical data, collected images, and drafted the paper. All authors reviewed and approved the final manuscript.

## CONSENT

Oral consent to treatment and written consent before each surgical procedure were obtained from the patient. Written permission to publish the patient's case and photographs was acquired.

## Data Availability

Data sharing is not applicable to this article as the paper is a case presentation ‐ no new data were created or analyzed in this study.
